# Non-Contact Monitoring of Breathing Pattern and Respiratory Rate via RGB Signal Measurement

**DOI:** 10.3390/s19122758

**Published:** 2019-06-19

**Authors:** Carlo Massaroni, Daniela Lo Presti, Domenico Formica, Sergio Silvestri, Emiliano Schena

**Affiliations:** 1 Unit of Measurements and Biomedical Instrumentation, Department of Engineering, Università Campus Bio-Medico di Roma, 00128 Rome, Italy; d.lopresti@unicampus.it (D.L.P.); s.silvestri@unicampus.it (S.S.); e.schena@unicampus.it (E.S.); 2 Unit of Neurophysiology and Neuroengineering of Human-Technology Interaction, Department of Engineering, Università Campus Bio-Medico di Roma, 00128 Rome, Italy; d.formica@unicampus.it

**Keywords:** measuring system, measurements, contactless, respiratory rate, breathing pattern

## Abstract

Among all the vital signs, respiratory rate remains the least measured in several scenarios, mainly due to the intrusiveness of the sensors usually adopted. For this reason, all contactless monitoring systems are gaining increasing attention in this field. In this paper, we present a measuring system for contactless measurement of the respiratory pattern and the extraction of breath-by-breath respiratory rate. The system consists of a laptop’s built-in RGB camera and an algorithm for post-processing of acquired video data. From the recording of the chest movements of a subject, the analysis of the pixel intensity changes yields a waveform indicating respiratory pattern. The proposed system has been tested on 12 volunteers, both males and females seated in front of the webcam, wearing both slim-fit and loose-fit t-shirts. The pressure-drop signal recorded at the level of nostrils with a head-mounted wearable device was used as reference respiratory pattern. The two methods have been compared in terms of mean of absolute error, standard error, and percentage error. Additionally, a Bland–Altman plot was used to investigate the bias between methods. Results show the ability of the system to record accurate values of respiratory rate, with both slim-fit and loose-fit clothing. The measuring system shows better performance on females. Bland–Altman analysis showed a bias of −0.01 breaths·min${}^{-1}$, with respiratory rate values between 10 and 43 breaths·min${}^{-1}$. Promising performance has been found in the preliminary tests simulating tachypnea.

## 1. Introduction

Accurate measurement of vital signs and physiological parameters, such as body temperature, pulse rate, blood pressure, and respiratory rate, plays a pivotal role in the healthcare sector and management of patients. Among these, the respiratory rate ($f_{R}$) is still considered the neglected vital sign in both the clinical practice and sports activity monitoring [1,2]. Temporal changes in the respiratory rate may indicate relevant variations of the physiological status of the subject, even better than other vital signs (e.g., pulse rate) [2] and it is found to be more discriminatory between stable and unstable patients than pulse rate [1].

In a clinical setting, the respiratory rate is an early indicator of physiological deterioration [3] and a predictor of potentially dangerous adverse events [1]. Indeed, respiratory rate is an important predictor of cardiac arrest and of unplanned intensive care unit admission [1], as well as an independent prognostic marker for risk assessment after acute myocardial infarction [4]. Besides, it is fundamental in the early detection and diagnosis of dangerous conditions such as sleep apnea [5], sudden infant death syndrome, chronic obstructive pulmonary disease, and respiratory depression in post-surgical patients [6]. In intensive care units, the respiratory waveform and $f_{R}$ are typically recorded. In mechanically ventilated patients, such data can be obtained directly by the mechanical ventilator traces [7] or retrieved by pulse oximetry sensors [8]. However, $f_{R}$ is typically collected at regular interval by operators (i.e., every 8–10 h) in the clinical setting outside this ward, while is often neglected in home monitored people and patient [1].

Conventional methods for measuring respiratory parameters require sensing elements in contact with the patient [9]. These methods are mainly based on the analysis of several parameters sampled from the inspiratory and/or expiratory flow. Differently, approaches based on the measurement of respiratory-related chest and abdominal movements have been also adopted [10]. Sensors may be directly attached on the torso [11] or integrated into clothing fibers. Several sensors have been used as resistive sensors, capacitive sensors, inductive sensors. Such monitoring systems must be worn and powered [11]. Additionally, they may cause undesirable skin irritation and discomfort, especially when long-term monitoring is required or during sleep. Substantial evidence indicates all these contact-based measurement techniques may influence the underlying physiological parameters being measured [12].

Contactless monitoring systems may overcome these issues related to placing sensors on patients and influence the measurand [13]. Mainly, solutions based on the analysis of depth changes of the torso using time-of-flight sensors [14] during breathing, low-power ultra wideband impulse radio radar [15,16], and laser Doppler vibrometers [17,18,19] have been designed and tested. Principal limitations of such solutions are related to the high cost of the instrumentation, need for specialized operators, and, in some cases, a low signal-to-noise ratio. Contactless monitoring systems based on the use of optical sensors are gaining preeminence in the field of respiratory monitoring mainly because of recent progress in video technology. Commercial and industrial cameras may be exciting solutions as they provide low-cost and easy-to-use non-contact approaches for measuring and monitoring physiological signals [4]. Some attempts have been made to record respiratory parameters from breathing-related movements of thoraco-abdominal area, face area, area at the edge of the shoulder, pit of the neck [20,21,22,23,24,25]. Then, different approaches have been also used to post-process the video to extract the respiratory-related signal mainly based on image subtraction [26], optical flow analysis [27], Eulerian Video Magnification [24] and Independent Component Analysis (ICA) applied to pixel intensity changes [28]. By the review of the literature, there is a lack of results about accuracy of such methods in the monitoring of eupneic respiratory pattern and $f_{R}$ monitoring, since the majority of the cited studies present proof of concepts and preliminary tests, but accuracy evaluation is not performed. When available, typically a frequency-domain analysis is carried out to extract the frequency content of the respiratory-related video signal and to measure the average respiratory rate. Since analysis with these techniques requires the recording of the torso movement, clothing can influence the data quality and validity of the methods. However, no studies have focused on such potential influences on respiratory pattern and $f_{R}$ measurement. Only a preliminary study of our research group tried to investigate this influencing factor in [29].

In this paper, we present a measuring system capable of non-contact monitoring of respiratory pattern by using RGB video signal acquired from a single built-in high-definition webcam. The aim of this study is three-fold: (i) the development of the measuring system and the related algorithm for the extraction of breath-by-breath $f_{R}$ values; (ii) the evaluation of the error between the breath-by-breath $f_{R}$ values retrieved by using the proposed measuring system and those recorded with a reference instrument; and (iii) the analysis of influence of clothing (i.e., slim-fit and loose-fit) and sex on the performance of the proposed method.

## 2. Measuring System

The proposed measuring system is composed of a hardware module (i.e., a built-in webcam) for video recording and an algorithm for (i) preprocessing of the video to obtain a respiratory signal, and (ii) event detection, segmentation and extraction of breath-by-breath $f_{R}$ values. The working principle of the method used to extract respiratory information from a video is explained in the following section.

### 2.1. Light Intensity Changes Caused by Respiration

Each video can be considered a series of *f* frames (i.e., polychromatic images), where *f* is the number of the frames collected. Each frame is an image composed of three images in the red (R), green (G) and blue (B) channels. Each image in the R, G and B channels is a matrix composed of pixels. The size of the matrix (of dimensions *x* along the *x*-axis , and *y* along the *y*-axis) depends on the resolution of the camera used for the data collection. Each pixel assumes a value representing the color light intensity: the value 0 means black, whereas the maximum value is the white. The numerical values of each pixel depend on the number of bytes used to represent a given R, G, B channel. When considering commercial 8-bit/channel cameras (24-bit for RGB colors), the maximum value is 2${}^{8}$ (i.e., 255 colors including zero).

When an object is recorded by a video, the pixel of each frame of the video assume an intensity level caused by the light reflected from the object over a two-dimensional grid of pixels. In the RGB color model separate intensity signals corresponding to each channel—$V_{R}\left(x,y,f\right)$,$V_{G}\left(x,y,f\right)$,$V_{B}\left(x,y,f\right)$—can be recorded at each frame *f*. The measured intensity of any reflected light (*V*) can be decomposed into two components: (i) intensity of illumination (*I*), and (ii) reflectance of the surface (*R*):

(1) V(x,y,f)=I(x,y,f)·R(x,y,f).

The respiratory activity causes the periodic movement of the chest wall. During inspiration, the ribcage widens: it results in an upward movement of the thorax; during expiration, the opposite occurs. By considering the chest wall covered by clothing as the surface framed by the camera, and the intensity of illumination almost constant, the changes of intensity of reflected light between two consecutive frames can be considered caused by the movement of the chest surface. Breathing-related chest movements are transmitted to the clothing (e.g., t-shirts, sweaters), so the subsequent changes of *V* can be used to collect respiratory patterns and events indirectly. Loose- or slim-fit clothing differently adhere to the skin. In the case of slim-fit clothing, we can hypothesize the complete transfer of chest wall movement to the side of the t-shirt framed by the camera, whereas only a partial transfer in the case of loose-fit clothing.

### 2.2. Hardware for Video Data Recording

**Figure 1:**
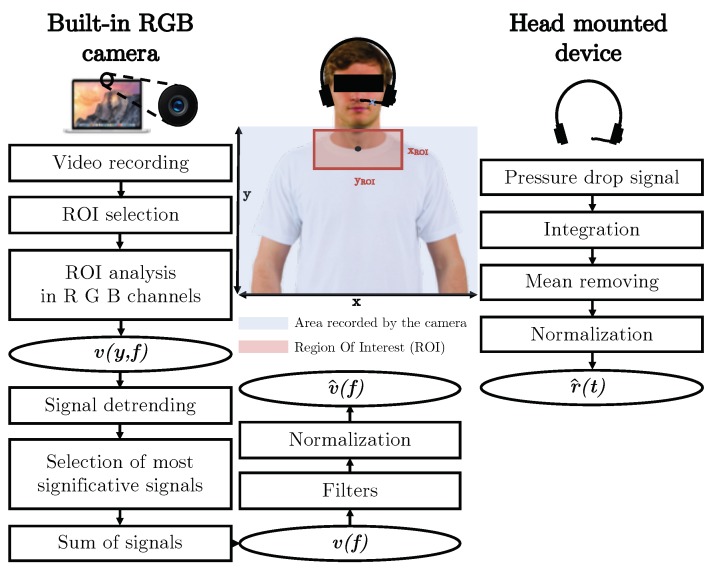
Flowchart presenting all the steps carried out to extract the respiratory pattern from video recorded with the built-in camera (on the left) and from the pressure-drop signal collected at the level of nostrils with the reference device (on the right). Region Of Interest (ROI) is the red rectangle; the area recorded by the camera is highlighted with the blue rectangle, the black point is the pixel (with coordinates $x_{P}$, $y_{P}$) at the level of the jugular notch.

The proposed system needs to collect a video of a person seated in front of the camera (Figure 1). The hardware module consists of a built-in CCD RGB webcam (iSight camera) integrated into a MacBook Pro laptop (by Apple Inc., California, USA). This camera is used to collect video with a resolution of 1280·720 pixel. Video images are recorded at 24-bit RGB with three channels, 8 bits per channel. A bespoke interface was developed in Matlab (MathWorks, Massachusetts, USA) to record the video and pre-process the data (i.e., images) collected with the camera. The video is collected for 120 s at a frame rate of 30 Hz, which is enough to register the breathing movements.

### 2.3. Algorithm for the Preprocessing of the Video

The preprocessing of the recorded video is performed off-line via a bespoke algorithm developed in Matlab, which is an upgraded version of the algorithm presented in our previous papers [29,30]. Several steps must be followed as shown in Figure 1.

Basically, after the video is loaded, the user (i.e., the one who is designated to analyze the data) is asked to select one pixel (with coordinates $x_{P}$, $y_{P}$) at the level of the jugular notch (i.e., the anatomical point near the suprasternal notch) in the first frame of the video. This anatomical marker has been chosen because it is easily identifiable (see Figure 1).

Automatically a rectangular region of interest (in short ROI) is delineated, with dimensions $x_{ROI}$ × $y_{ROI}$:

(2) $$\begin{array}{c} x_{ROI}=\left[x_{P}-\frac{1}{100}\;\cdot \;15\;\cdot \;x,x_{P}+\frac{1}{100}\;\cdot \;15\;\cdot \;x\right],\\ y_{ROI}=\left[y_{P}-\frac{1}{100}\;\cdot \;15\;\cdot \;y,y_{P}+\frac{1}{100}\;\cdot \;15\;\cdot \;y\right], \end{array}$$

where *x* and *y* are the *x*-axis and *y*-axis frame dimensions (related to camera resolution), respectively.

The selected ROI is then split into three same-size images corresponding to the red, green, and blue channels. At each frame *f*, the intensity components of each channel I(x,y,c,f) are obtained, where *c* is the color channel (i.e., red (R), green (G), and blue (B)). Then, the intensity components are averaged for each line *y* of the ROI according to Equation (3):

(3) $$v\left(y,f\right)=\frac{1}{x_{ROI}}\;\cdot \;\sum_{x=1}^{x_{ROI}}\left(\sum_{c=R,G,B}I\left(x,y,c,f\right)\right),$$

where $y\in y_{ROI}$.

From each v(y,f), the mean of the signal is removed from the signal itself (i.e., the signal is detrended). The standard deviation of each v(y,f) signal is then calculated. The 5% of the v(y,f) with the higher standard deviations are selected. The 5% value was selected with an empirical approach using data from previous experiments carried out on volunteers aimed at calibrating the algorithm. The 5% of the v(y,f) are used to calculate the mean value considering the selected lines at each frame. The v(f) signal is obtained with this procedure. At that point, filters were applied to the v(f) signal. For filtering the signal and to emphasize the respiratory content, adequate cut-off frequencies and bandwidth need to be defined. A bandpass configuration was chosen, by fixing the low cut-off frequency around 0.05 Hz, to avoid the slow signal variations unrelated to respiratory movements and a high cut-off frequency around 2 Hz. In this way, the changes generated by the respiratory movements recorded to the webcam sensor can be adequately isolated and relayed to the subsequent elaboration stages. A third order Butterworth digital filter was employed. Finally, the v(f) signal is normalized to obtain $\hat{v}\left(f\right)$ as reported in the following Equation (4):

(4) $$\hat{v}\left(f\right)=\frac{v(f)-\mu (v(f))}{\sigma (v(f))}$$

where μ(v(f)) and σ(v(f)) are the mean and standard deviation of signal v(f), respectively.

**Figure 2:**
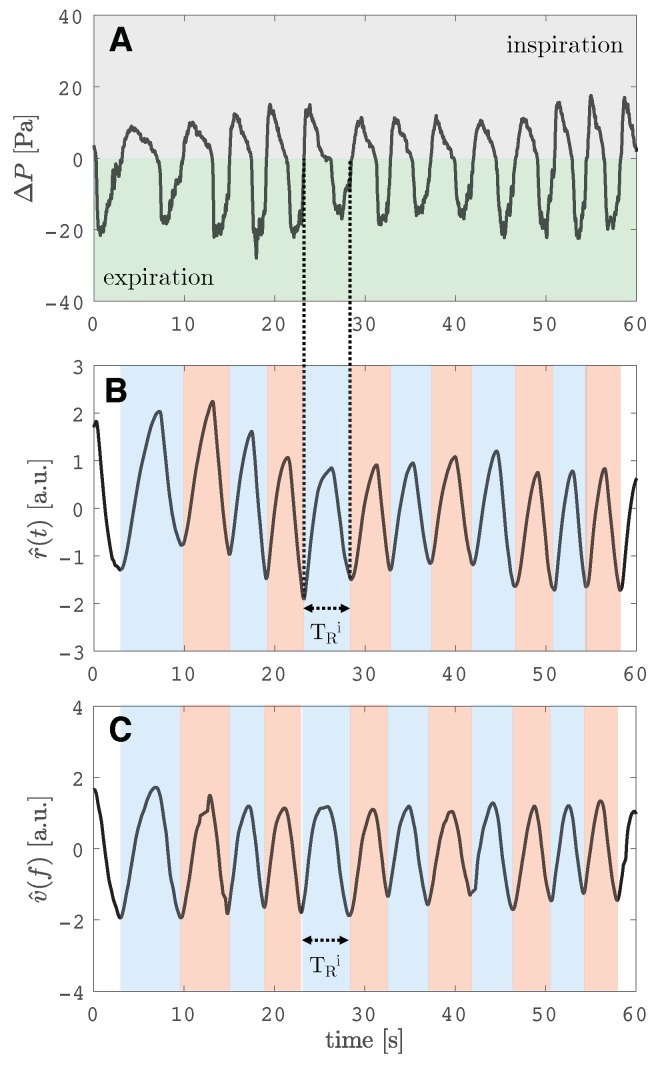
(**A**) ΔP signal recorded by the reference instrument at the level of the nostrils. In grey, the signal collected during the inspiration (positive pressure), while in green, the signal recorded during the expiration (negative pressure). (**B**) Reference respiratory pattern signal ($\hat{r}\left(t\right)$) obtained from data processing of ΔP signal. (**C**) Respiratory pattern signal obtained from the proposed measuring system ($\hat{v}\left(f\right)$). Figure in (**B**) and (**C**) show similar patterns: during the inspiratory phase the signal increases, while during the expiratory phase they decrease. The duration of one breath ($T_{R}\left(i\right)$) is shown on both the $\hat{r}\left(t\right)$ and $\hat{v}\left(f\right)$ signals.

The signal $\hat{v}\left(f\right)$ is used for extracting respiratory temporal information (i.e., period duration—$T_{R}$ and respiratory rate—$f_{R}$) since $\hat{v}\left(f\right)$ would be proportional to the changes in the intensity component, and thus to the underlying respiratory signal of interest (Figure 2). A window of 60 s is shown in Figure 2B. In this figure the apnea phase of about 5 s used for synchronizing reference signal and video-derived signal in the experimental trials is not shown (see Section 3.1).

## 3. Tests and Experimental Trials

### 3.1. Participants and Tests

In this study, we enrolled 12 participants (6 males and 6 females) with a mean age 24 ± 4 years old, mean height of 165 ± 15 cm, mean body mass of 60 ± 10 kg). All the participants provided informed consent. We have created a data set for evaluation of the proposed system. We aim to cover normal breathing (i.e., respiratory frequency in the range 8–25 breaths·min${}^{-1}$), abnormal breathing (i.e., tachypnea) and apnea stages.

Each participant was invited to sit on a chair in front of the web camera at a distance of about 1.2 m. The user adjusted the screen of the laptop in order to record the trunk area (as shown in Figure 1). All the experiments were carried out indoor (in a laboratory room) and with a stable amount of light delivered by neon lights and three windows as sources of illumination. The participants’ shoulders were turned towards the furnishings of the room. The windows were lateral to the scene recorded by the camera. Other people were in the room during the data collection but not allowed to pass near the shooting area.

Participants were asked to keep still and seated, and to breathe spontaneously by facing the webcam. Each volunteer was called to breathe quietly for around 5 s, simulate an apnea of duration <10 s, and then to breathe quietly at self-paced $f_{R}$ for all the duration of the trial (120 s). Each volunteer carried out two trials with the same experimental design: in the first trial, the participant wore a loose-fit t-shirt; in the second trials, a slim-fit t-shirt. Two volunteers were also invited to simulate abnormal breathing (i.e., tachypnea) that is characterized by high $f_{R}$ values (>35 bpm).

At the same time, respiratory pattern was recorded with a reference instrument described in the following Section 3.2.

### 3.2. Reference Instrument and Signal

For registering reference pattern, a head-mounted wearable device was used. We already used this system in a similar scenario [31]. This device is based on the recording of the pressure-drop (ΔP) that occurs during the expiratory/inspiratory phases of respiration at the level of nostrils. The device consists of a cannula attached to the jaw with tape: one piece of tape at the end of the nostrils in order to collect part of the nasal flow while the other tap is connected to a static tap of a differential digital pressure sensor (i.e., Sensirion—model SDP610, pressure range up to ±125 Pa). The pressure data were recorded with a dedicated printed circuit board described in [31], at 100 Hz of sample rate. Data were sent to a remote laptop via a wireless connection and archived.

Negative pressure was collected during the expiratory phase and positive pressure during the inspiratory phase, as can be seen in Figure 2A. Then, a temporal standard cumulative trapezoidal numerical integration of the ΔP signal was carried out to obtain a smooth respiratory signal for further analysis (r(t)) and to emphasize the maximum and minimum peaks. Afterward, such integrated r(t) has been filtered using a bandpass Butterworth filter in the frequency range 0.05–2 Hz and normalized as in Equation (4) and $\hat{r}\left(t\right)$ has been obtained. This $\hat{r}\left(t\right)$ is the reference respiratory pattern signal, then used to extract breath-by-breath $f_{R}$ reference values (i.e., $f_{R}\left(i\right)$).

As shown in Figure 2B, one breath is the portion of the signal between the starting point of the inspiration and the end of the following expiration. During the inspiratory phase, the ΔP signal pass from 0 to positive values (grey area in Figure 2A), and r(t) is an increasing signal. During the expiratory phase, the opposite situation: ΔP signal passes from 0 to negative values (green area in Figure 2A), and $\hat{r}\left(t\right)$ is a decreasing signal.

### 3.3. Respiratory Rate Calculation

The breathing rate can be extracted from both the reference signal $\hat{r}\left(t\right)$ and $\hat{v}\left(f\right)$ either in the frequency or time domains [21,32]. The analysis in the time domain requires the identification of specific points on the signal. Mainly, two different approaches may be used: (i) based on the identification of the maximum and minimum points; or (ii) the zero-crossing point individuation on the signals. In this work, we used a zero-crossing-based algorithm. We used the same algorithm for the event detection on both the reference signal $\hat{r}\left(t\right)$ and $\hat{v}\left(f\right)$. The algorithm provides the detection of the zero-crossing points on the signal based on signum function. It allows determining the onset of each respiratory cycle, characterized by a positive going zero-crossing value. The signum function of a real number *x* is defined as in the following Equation (5):

(5) $$sgn\left(x\right):\left\{\begin{array}{cc} -1 & \mathrm{if}x_{i}<0,\\ 0 & \mathrm{if}x_{i}=0,\\ 1 & \mathrm{if}x_{i}>0, \end{array}\right.$$

where $x_{i}$ is the value *x* of the signal for frame index *i* corresponding to the onset of a respiratory cycle. Then, the algorithm provides the location of local minimum points on the signal and their indices between respiratory cycle onsets determined in the first step.

The duration of each *i*-th breath—$T_{R}\left(i\right)$—is then calculated as the time elapsed between two consecutive minima points (expressed in s). Consequently, the *i*-th breath-by-breath breathing rate $f_{R}\left(i\right)$, expressed in breaths per minute (bpm), is calculated as in Equation (6):

(6) $$f_{R}\left(i\right)=\frac{60}{T_{R}\left(i\right)}.$$

### 3.4. Data Analysis

We recorded the breath-by-breath respiratory rate with our system and the reference instrument and evaluated the discrepancies coming from their comparison. Signals obtained from the measuring system have been compared to the reference signals. Firstly the $\hat{r}\left(t\right)$ and $\hat{v}\left(f\right)$ were synchronized to be directly compared. We used the apnea stage to detect a common event on both signals. All the analysis were carried out on both the $\hat{r}\left(t\right)$ and $\hat{v}\left(f\right)$ that occur after the first end expiratory point after the apnea stage. The breath-by-breath $f_{R}$ values have been compared between instruments by extracting such values with the time-domain analysis from $\hat{r}\left(t\right)$ (i.e., $f_{R}\left(i\right)$) and $\hat{v}\left(f\right)$ (i.e., ${\hat{f}}_{R}\left(i\right)$).

To compare the values gathered by the reference instrument and computed by the video-based method, we use the mean absolute error (MAE) as in Equation (7):

(7) $$MAE=\frac{1}{n}\;\cdot \;\sum_{i=1}^{n}\left|{\hat{f}}_{R}\left(i\right)-f_{R}\left(i\right)\right|,$$

where *n* is the number of breaths recognized by the algorithm for each subject in the trial.

Then, the standard error of the mean (SE) is calculated as in Equation (8):

(8) $$SE=\frac{SD}{\sqrt{n}},$$

where SD is the standard deviation of the absolute difference between estimations and reference data ${\hat{f}}_{R}\left(i\right)-f_{R}\left(i\right)$. Standard error was used to provide a simple estimation of uncertainty.

Lastly, the percentage difference between instruments was calculated as in Equation (9), per each volunteer:

(9) $$\%E=\frac{1}{n}\;\cdot \;\sum_{n}\frac{{\hat{f}}_{R}\left(i\right)-f_{R}\left(i\right)}{f_{R}\left(i\right)}\;\cdot \;100.$$

Additionally, we used the Bland–Altman analysis to investigate the agreement between the proposed method and the reference, in the whole range of $f_{R}$ measurement. With this graphical method we investigated if the differences between the two techniques against the averages of the two techniques presented a tendency at the different $f_{R}$ collected during the trials. The Bland–Altman analysis was used to obtain the mean of the Differences (MOD) and the limits of Agreements (LOAs) values [33] that are typically reported in other studies and extremely useful when comparing our results with the relevant scientific literature [2].

To fulfill the scope of this paper we carried out three separate analyses using these metrics for comparisons. Firstly, we used the data collected with slim-fit and loose-fit clothing to investigate the influence of clothing on the performance of the proposed method, using both male and female data. Then, we separately use the data collected from male and from female to investigate the influence of sex on performance. Lastly, the overall performance of the proposed measuring system has been tested considering all the breath-by-breath $f_{R}$ (*n* = 411). Preliminary tests have been also done using data collected from two volunteers during tachypnea.

## 4. Experimental Results

The detection of apnea stages used for synchronizing the signals on $\hat{r}\left(t\right)$ and $\hat{v}\left(f\right)$ was always possible. Therefore, no trials were excluded from the analysis. During the apnea, the signal collected by the reference instrument is a constant and null ΔP; constant signals were also found in $\hat{v}\left(f\right)$.

**Figure 3:**
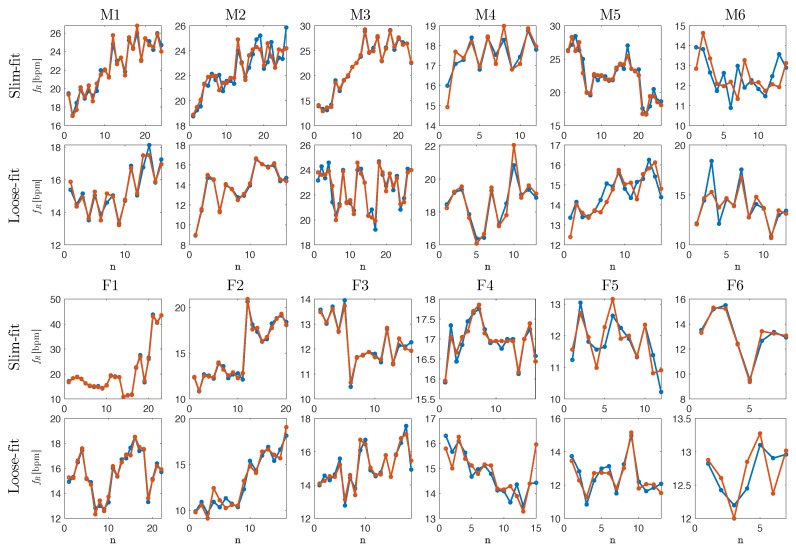
Breath-by-breath values of the respiratory rate collected by the reference system (blue dots and lines) and by the proposed measuring system (orange dots and lines). Data from male (i.e., M1, M2, M3, M4, M5, M6) and female (F1, F2, F3, F4, F5, F6) volunteers wearing slim-fit and loose-fit clothing are reported. Details about MAE, SE and %E values per each volunteer with slim-fit and loose-fit clothing are reported in Table 1.

Table 1 summaries the number of breaths, average ${\hat{f}}_{R}$ and $f_{R}$ values, MAE, SE and %E for each subject, at the two t-shirt fittings. MAE value was always lower than 0.78 bpm, while standard error was <0.24 bpm in all the volunteers. %E values were both negative and positive: the maximum value was 0.62%. The performance of the proposed method in the measurement of breath-by-breath respiratory frequencies can be appreciated in Figure 3.

### 4.1. Influence of Clothing Type

**Figure 4:**
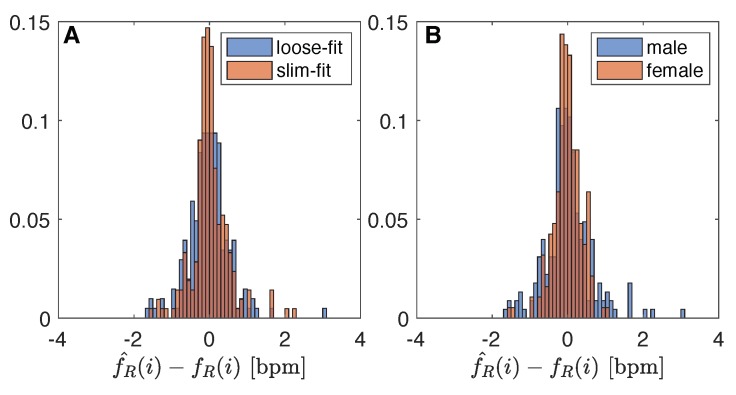
Difference distribution: (**A**) influence of clothing type (i.e., slim-fit vs loose-fit); (**B**) influence of sex (i.e., male vs female).

The influence of clothing was investigated by analyzing the difference (i.e., $\hat{f_{R}}\left(i\right)-f_{R}\left(i\right)$) distribution considering all the data obtained from male and female together. Since the sample size and bin width of the histograms are different between slim-fit (*n* = 211) and loose-fit (*n* = 203) data, it is difficult to compare them. So, we normalize the histograms so that all the bar heights add to 1, and we use a uniform bin width (0.1 bpm). With the slim-fit clothing, the 28% of the differences between the two instruments were in the range ±0.1 bpm (94% of data in the range ±1 bpm), while with the loose-fit clothing only 19% of data (94% of data in the range ±1 bpm). For details refers to Figure 4A. The Bland–Altman showed a bias of −0.02 ± 1.07 bpm and 0.01 ± 0.98 bpm in the case of loose-fit and slim-fit clothing, respectively. From the Bland–Altman plot, neither proportional error nor magnitude of measurements dependence were found.

### 4.2. Influence of Sex

**Figure 5:**
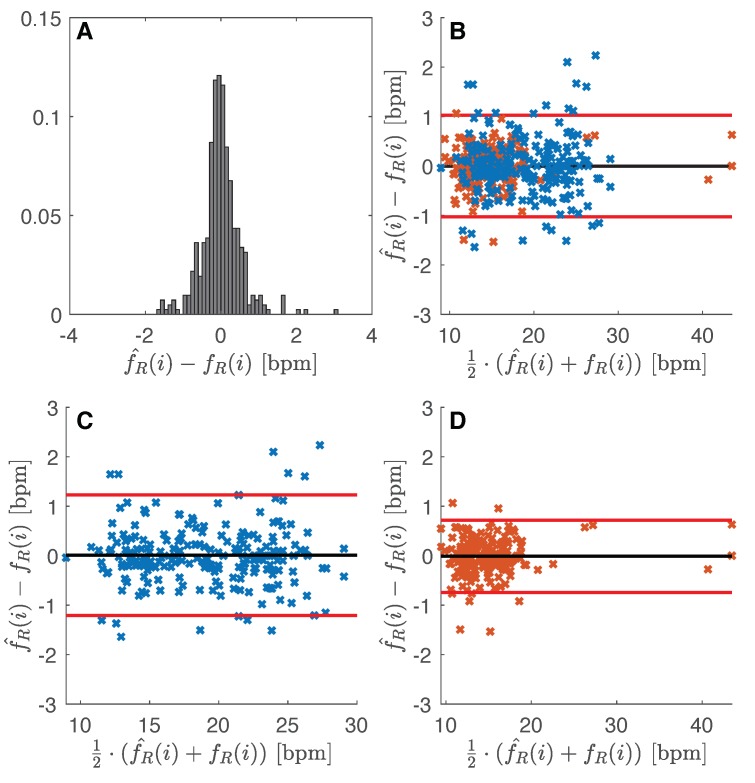
(**A**) Difference distribution between proposed method and reference respiratory rate values; (**B**) Bland–Altman plot obtained considering all the data recorded by male and female volunteers: black line is the MOD, red lines are the LOAs (i.e., ±1.96 times the standard deviation); (**C**,**B**) Bland–Altman plot obtained considering data recorded by male volunteers; (**D**) Bland–Altman plot obtained considering data recorded by female volunteers.

The influence of sex on the performance of the measuring system was investigated by analyzing the difference ($\hat{f_{R}}\left(i\right)-f_{R}\left(i\right)$) distribution considering all the data obtained from data collection carried out with slim-fit and loose-fit clothing. Normalized histograms with uniform bin widths (0.1 bpm) were used since the difference sample size between male data (*n* = 226) and female data (*n* = 188). In the male group, 21% of data show difference between instrument in the range ±0.1 bpm (90% of data in the range ±1 bpm), while in the female group was 27% of the data (98% of data in the range ±1 bpm). Figure 4B shows the two distributions. The Bland–Altman analysis revealed a bias of 0.01 ± 1.22 bpm (see Figure 5C) and −0.01 ± 0.73 bpm (see Figure 5D) for male and female volunteers, respectively. All the $f_{R}$ values recorded by the male volunteers are between 10 and 30 bpm (mean 19.14 bpm, SD 4.55 bpm). In female volunteers, five $f_{R}$ values over 25 bpm can be observed in Figure 5D, while 96% of the data are in the range of 10–20 bpm (mean 14.99 bpm, SD 4.47). Bland–Altman analysis shows the absence of proportional error and magnitude of measurements dependence.

### 4.3. Overall Performance

All the $f_{R}\left(i\right)$ extracted from $\hat{r}\left(t\right)$ and $\hat{v}\left(f\right)$ per each subject with slim-fit and loose-fit clothing are presented in Figure 3. Data extracted from signal collected with the proposed measuring system follow the data extracted from reference signal in each subject, both a low $f_{R}$ and high $f_{R}$. Similar variations in $f_{R}$ estimates can be clearly observed in that figure.

Figure 5A shows the difference distribution of all the 414 breaths collected: the 24% of the differences are in the interval of ±0.1 bpm, and only 6% of data shows differences higher than ±1 bpm. Bland–Altman analysis (Figure 5B) demonstrates a bias with a MOD close to 0 (i.e., −0.01 bpm) and LOAs of 1.02 bpm. Bland–Altman analysis allows us to assess the absence of proportional error and magnitude of measurements dependence.

### 4.4. Preliminary Results during Tachypnea

**Figure 6:**
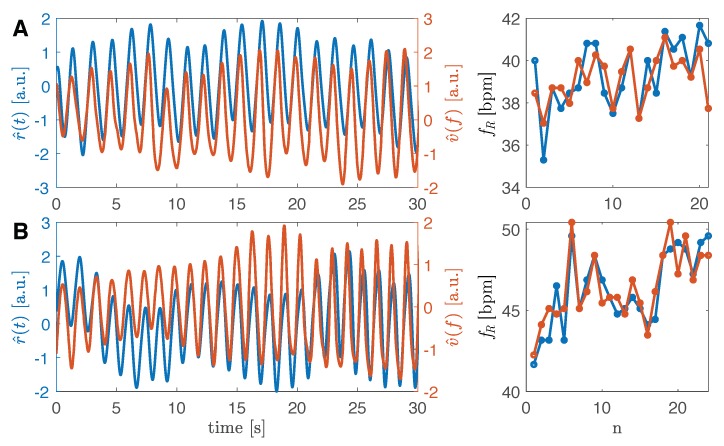
Two patterns collected from two volunteers simulating tachypnea. In blue, the reference signal $\hat{r}\left(t\right)$, in orange the $\hat{v}\left(f\right)$ signal. In the first volunteer (**graphs A**), the average ${\hat{f}}_{R}$ is 39.14 bpm while the average $f_{R}$ is 39.34 bpm. In the second volunteer (**graphs B**), the average ${\hat{f}}_{R}$ is 46.44 bpm while the average $f_{R}$ is 46.28 bpm.

The proposed measuring system has also been preliminarily tested on two subjects during tachypnea. Figure 6 reports two examples of 30 s data collection on two volunteers. By applying the algorithm for the $f_{R}$ calculation, we found a MAE of 1.05 bpm, a SE 0.13 bpm and a %E of −0.24% for the first volunteer; second volunteer data show a MAE of 0.48 bpm, SE of 0.08 bpm and %E of 0.04%. Due to the small sample size, Bland–Altman was not used to summarize bias between methods.

## 5. Discussion

In this paper, a single built-in camera system is proposed for the extraction of the respiratory pattern and the estimation of breath-by-breath $f_{R}$. The built-in camera of a commercial laptop allows the non-intrusive, ecological, and low-cost recording of chest wall movement. The algorithm for the processing of images allows (i) the chest wall video recording at sufficient frame rate (i.e., 30 Hz), (ii) the selection of a pixel for further semi-automatic selection of a ROI for the measurement of the pixel intensity change, in order to extract video-based respiratory pattern $\hat{v}\left(f\right)$, and (iii) the post-processing of the $\hat{v}\left(f\right)$ signal to estimate breath-by-breath $f_{R}$ values. The proposed system has been tested on healthy participants. Tests were carried out on male and female participants wearing both slim-fit and loose-fit t-shirts to simulate real respiratory monitoring conditions (e.g., a subject at home, patient in a medical room, etc.). In the literature, rarely authors take into account the influence of sex and clothing when camera-based methods are used. Additionally, in this paper, we used an unobtrusive head-mounted wearable as reference instrument to not compromise the area recorded by the camera.

Signals obtained with the proposed method allow clear identification of the apnea stages, breathing pattern at quiet pace and during tachypnea in all the trials. Considering the breath-by-breath $f_{R}\left(i\right)$ values, we obtained comparable MAE and SE values in the two groups (slim-fit vs. loose-fit). From the analysis of the bias revealed by the Bland–Altman plots, we found slightly better results with volunteers wearing slim-fit clothing (LOAs of ±0.98 bpm against ±1.07 bpm with loose-fit clothing). These results confirm those obtained in [29]. Considering the sex, results demonstrated good performance with both males and females with slightly lower bias in females (−0.01 ± 0.73 bpm) than in males (0.01 ± 1.22 bpm). By considering all 414 breaths, the Bland–Altman analysis demonstrates a bias of −0.01 ± 1.02 bpm of the proposed method when compared to the $f_{R}$ values gathered by the reference instrument. The method proposed in [20] achieves bias of −0.32±1.61 bpm when tested in similar setting and participants. Then, the bias we found is comparable with the one reported in [34] (i.e., −0.02 ± 0.83 bpm) where the pseudo-Wigner–Villetime frequency analysis was used (with a $f_{R}$ resolution of 0.7324 bpm). The performances we obtained are better than those obtained in [35] where the average $f_{R}$ were considered (bias of 0.37 ± 1.04 bpm), and advanced signal and video processing techniques, including developing video magnification, complete ensemble empirical mode decomposition with adaptive noise, and canonical correlation analysis were used in the post-processing phase. When compared to depth sensors used on participant in supine position [16], our method demonstrates comparable results with simplicity and cost (∼0.01 ± 0.96 bpm in [16]). Despite the absence of contact with the subject, the proposed method shows overall performance similar to those obtained with wearable device for $f_{R}$ monitoring requiring direct contact with the torso (e.g., garment with optical fibers showed bias of −0.02 ± 2.04 bpm in [36], during quiet breathing). In contrast to other research studies, we did not use a background behind the user to test the system in conditions resembling real application scenarios. Further tests might be focused on extracting respiratory volumes by using a more structured environment during video collection as in [37].

One of the main limitations of this study is the limited number of subjects included in the analysis. For this reason, we did not perform any statistical analysis because population size does not allow any statistically significative conclusions. Additionally, we tested the proposed method at one distance between camera and subject (i.e., 1.2 m).

Further effort will be mainly devoted to addressing these points. Tests will be carried out to investigate the performance of the system in different scenarios at different subject–camera relative distances, and on many subjects. Furthermore, performance of the method will be tested in a wide range of atypical respiratory pattern (i.e., tachypnea, deep breaths, Cheyne-Stokes) and in extracting additional respiratory parameters (e.g., duration of expiratory and inspiratory parameters, inter-breathing variations). We are already testing the validity of additional techniques based on pixel flow analysis to remove unrelated breathing movements. Additionally, we are working on feature selection approaches to use the proposed method for respiratory monitoring when small movements of the user happen. We hope to use the proposed measuring system for respiratory monitoring even with undesired subject’ motion, also by implementing a fully automatic process to detect ROI from video frames. These steps will allow automatic and long-term data collection.

## Figures and Tables

**Table 1:** Breath-by-breath analysis: average $\hat{f_{R}}$, average $f_{R}$, mae, se and %e values per each volunteer at the different t-shirt fitting. MAE: Mean Absolute Error ; SE: Standard Error of the mean.

Vol.	T-Shirt	#	$\hat{f_{R}}$	$f_{R}$	MAE	SE	%E
	Fitting	Breaths	[bpm]	[bpm]	[bpm]	[bpm]	[%]
M1	slim	24	22.29	22.28	0.76	0.18	−0.52
	loose	16	15.33	15.31	0.27	0.05	0.18
M2	slim	17	13.90	13.86	0.58	0.09	0.46
	loose	26	22.48	22.39	0.14	0.02	0.28
M3	slim	23	22.21	22.18	0.27	0.04	0.14
	loose	27	22.55	22.55	0.35	0.0	0.04
M4	slim	12	17.53	17.55	0.32	0.09	0.19
	loose	13	18.57	18.52	0.33	0.09	−0.16
M5	slim	26	22.32	22.46	0.65	0.13	−0.52
	loose	16	14.51	14.60	0.43	0.08	−0.62
M6	slim	14	12.49	12.51	0.78	0.14	0.21
	loose	13	13.82	13.92	0.60	0.24	−0.10
F1	slim	23	20.62	20.67	0.24	0.04	−0.13
	loose	22	15.61	15.57	0.23	0.03	0.26
F2	slim	20	15.08	15.07	0.27	0.04	0.14
	loose	16	13.14	13.07	0.60	0.10	0.55
F3	slim	16	12.27	12.30	0.11	0.02	−0.15
	loose	19	15.08	15.06	0.25	0.04	0.18
F4	slim	17	16.96	16.95	0.11	0.02	0.07
	loose	15	14.85	14.78	0.37	0.10	0.53
F5	slim	12	11.83	11.78	0.35	0.07	0.50
	loose	13	12.50	12.55	0.36	0.04	−0.32
F6	slim	8	13.16	13.14	0.23	0.08	0.17
	loose	7	12.71	12.69	0.23	0.07	0.16
Overall	-	414	-	-	0.39	0.02	0.07

## References

[1] Cretikos M.A., Bellomo R., Hillman K., Chen J., Finfer S., Flabouris A. (2008). Respiratory rate: The neglected vital sign. Med. J. Aust..

[2] Nicolò A., Massaroni C., Passfield L. (2017). Respiratory frequency during exercise: The neglected physiological measure. Front. Physiol..

[3] Smith I., Mackay J., Fahrid N., Krucheck D. (2011). Respiratory rate measurement: A comparison of methods. Br. J. Healthc. Assist..

[4] Barthel P., Wensel R., Bauer A., Müller A., Wolf P., Ulm K., Huster K.M., Francis D.P., Malik M., Schmidt G. (2012). Respiratory rate predicts outcome after acute myocardial infarction: A prospective cohort study. Eur. Heart J..

[5] Younes M. (2008). Role of respiratory control mechanisms in the pathogenesis of obstructive sleep disorders. J. Appl. Physiol..

[6] Rantonen T., Jalonen J., Grönlund J., Antila K., Southall D., Välimäki I. (1998). Increased amplitude modulation of continuous respiration precedes sudden infant death syndrome: Detection by spectral estimation of respirogram. Early Hum. Dev..

[7] Schena E., Massaroni C., Saccomandi P., Cecchini S. (2015). Flow measurement in mechanical ventilation: A review. Med. Eng. Phys..

[8] Brochard L., Martin G.S., Blanch L., Pelosi P., Belda F.J., Jubran A., Gattinoni L., Mancebo J., Ranieri V.M., Richard J.C.M. (2012). Clinical review: Respiratory monitoring in the ICU-a consensus of 16. Crit. Care.

[9] Massaroni C., Nicolò A., Lo Presti D., Sacchetti M., Silvestri S., Schena E. (2019). Contact-based methods for measuring respiratory rate. Sensors.

[10] Massaroni C., Di Tocco J., Presti D.L., Longo U.G., Miccinilli S., Sterzi S., Formica D., Saccomandi P., Schena E. (2019). Smart textile based on piezoresistive sensing elements for respiratory monitoring. IEEE Sens. J..

[11] Dionisi A., Marioli D., Sardini E., Serpelloni M. (2016). Autonomous wearable system for vital signs measurement with energy-harvesting module. IEEE Trans. Instrum. Meas..

[12] Gilbert R., Auchincloss J., Brodsky J., Boden W.A. (1972). Changes in tidal volume, frequency, and ventilation induced by their measurement. J. Appl. Physiol..

[13] Al-Naji A., Gibson K., Lee S.H., Chahl J. (2017). Monitoring of cardiorespiratory signal: Principles of remote measurements and review of methods. IEEE Access.

[14] Deng F., Dong J., Wang X., Fang Y., Liu Y., Yu Z., Liu J., Chen F. (2018). Design and Implementation of a Noncontact Sleep Monitoring System Using Infrared Cameras and Motion Sensor. IEEE Trans. Instrum. Meas..

[15] Lai J.C.Y., Xu Y., Gunawan E., Chua E.C., Maskooki A., Guan Y.L., Low K., Soh C.B., Poh C. (2011). Wireless Sensing of Human Respiratory Parameters by Low-Power Ultrawideband Impulse Radio Radar. IEEE Trans. Instrum. Meas..

[16] Bernacchia N., Scalise L., Casacanditella L., Ercoli I., Marchionni P., Tomasini E.P. Non contact measurement of heart and respiration rates based on Kinect™. Proceedings of the 2014 IEEE International Symposium on Medical Measurements and Applications (MeMeA).

[17] Marchionni P., Scalise L., Ercoli I., Tomasini E. (2013). An optical measurement method for the simultaneous assessment of respiration and heart rates in preterm infants. Rev. Sci. Instrum..

[18] Scalise L., Ercoli I., Marchionni P., Tomasini E.P. Measurement of respiration rate in preterm infants by laser Doppler vibrometry. Proceedings of the 2011 IEEE International Workshop on Medical Measurements and Applications Proceedings (MeMeA).

[19] Sirevaag E.J., Casaccia S., Richter E.A., O’Sullivan J.A., Scalise L., Rohrbaugh J.W. (2016). Cardiorespiratory interactions: Noncontact assessment using laser Doppler vibrometry. Psychophysiology.

[20] Lin K.Y., Chen D.Y., Tsai W.J. (2016). Image-Based Motion-Tolerant Remote Respiratory Rate Evaluation. IEEE Sens. J..

[21] Massaroni C., Lopes D.S., Lo Presti D., Schena E., Silvestri S. (2018). Contactless Monitoring of Breathing Patterns and Respiratory Rate at the Pit of the Neck: A Single Camera Approach. J. Sens..

[22] Bartula M., Tigges T., Muehlsteff J. Camera-based system for contactless monitoring of respiration. Proceedings of the 2013 35th Annual International Conference of the IEEE Engineering in Medicine and Biology Society (EMBC).

[23] Koolen N., Decroupet O., Dereymaeker A., Jansen K., Vervisch J., Matic V., Vanrumste B., Naulaers G., Van Huffel S., De Vos M. Automated Respiration Detection from Neonatal Video Data. Proceedings of the International Conference on Pattern Recognition Applications and Methods ICPRAM.

[24] Antognoli L., Marchionni P., Nobile S., Carnielli V., Scalise L. Assessment of cardio-respiratory rates by non-invasive measurement methods in hospitalized preterm neonates. Proceedings of the 2018 IEEE International Symposium on Medical Measurements and Applications (MeMeA).

[25] Bernacchia N., Marchionni P., Ercoli I., Scalise L. (2015). Non-contact measurement of the heart rate by a image sensor. Sensors.

[26] Bai Y.W., Li W.T., Chen Y.W. Design and implementation of an embedded monitor system for detection of a patient’s breath by double Webcams in the dark. Proceedings of the 12th IEEE International Conference on e-Health Networking Applications and Services (Healthcom).

[27] Janssen R., Wang W., Moço A., De Haan G. (2015). Video-based respiration monitoring with automatic region of interest detection. Physiol. Meas..

[28] Poh M.Z., McDuff D.J., Picard R.W. (2011). Advancements in noncontact, multiparameter physiological measurements using a webcam. IEEE Trans. Biomed. Eng..

[29] Massaroni C., Schena E., Silvestri S., Taffoni F., Merone M. Measurement system based on RBG camera signal for contactless breathing pattern and respiratory rate monitoring. Proceedings of the 2018 IEEE International Symposium on Medical Measurements and Applications (MeMeA).

[30] Massaroni C., Nicolò A., Girardi M., La Camera A., Schena E., Sacchetti M., Silvestri S., Taffoni F. (2019). Validation of a wearable device and an algorithm for respiratory monitoring during exercise. IEEE Sens. J..

[31] Taffoni F., Rivera D., La Camera A., Nicolò A., Velasco J.R., Massaroni C. (2018). A Wearable System for Real-Time Continuous Monitoring of Physical Activity. J. Healthc. Eng..

[32] Welch P. (1967). The use of fast Fourier transform for the estimation of power spectra: A method based on time averaging over short, modified periodograms. IEEE Trans. Audio Electroacoust..

[33] Altman D.G., Bland J.M. (1983). Measurement in medicine: The analysis of method comparison studies. Statistician.

[34] Reyes B.A., Reljin N., Kong Y., Nam Y., Chon K.H. (2017). Tidal Volume and Instantaneous Respiration Rate Estimation using a Volumetric Surrogate Signal Acquired via a Smartphone Camera. IEEE J. Biomed. Health Inform..

[35] Al-Naji A., Chahl J. (2017). Simultaneous tracking of cardiorespiratory signals for multiple persons using a machine vision system with noise artifact removal. IEEE J. Translat. Eng. Health Med..

[36] Massaroni C., Venanzi C., Silvatti A.P., Lo Presti D., Saccomandi P., Formica D., Giurazza F., Caponero M.A., Schena E. (2018). Smart textile for respiratory monitoring and thoraco-abdominal motion pattern evaluation. J. Biophotonics.

[37] Liu C., Yang Y., Tsow F., Shao D., Tao N. (2017). Noncontact spirometry with a webcam. J. Biomed. Opt..

